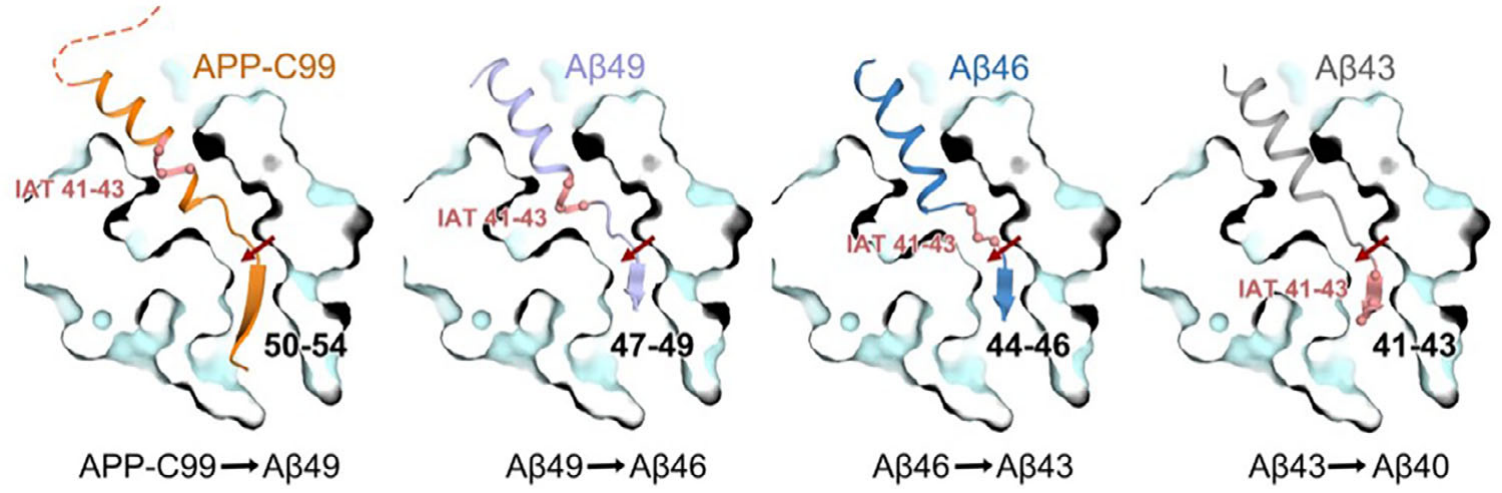# Molecular Mechanism of Substrate Recognition and Cleavage by Human γ‐Secretase

**DOI:** 10.1002/alz.092188

**Published:** 2025-01-03

**Authors:** Xuefei Guo

**Affiliations:** ^1^ Tsinghua university, Beijing, Beijing China

## Abstract

**Background:**

Successive cleavages of amyloid precursor protein C‐terminal 99 residues (APP‐C99) by human γ‐secretase result in amyloid‐β peptides (Aβs) of varying lengths, the main constituents of amyloid plaques in Alzheimer’s disease patients. Most cleavages have a step size of three residues, as exemplified by sequential generation of Aβ49, Aβ46, Aβ43, and Aβ40.

**Method:**

To elucidate the mechanism of substrate cleavage, we determined atomic structures of human γ‐secretase bound individually to APP‐C99, Aβ49, Aβ46, and Aβ43.

**Result:**

Remarkably, in all cases, the substrate has the same structural features: a transmembrane α‐helix that binds PS1, a three‐residue linker, and a β‐strand that forms a hybrid β‐sheet with two β‐strands of PS1. Proteolytic cleavage occurs at the peptide bond just preceding the substrate β‐strand.

**Conclusion:**

Therefore, each cleavage is followed by unwinding of the substrate α‐helix by one turn, translocation of the α‐helix towards the C‐terminus, and formation of a new β‐strand. This mechanism is consistent with existing biochemical data and may also explain the cleavages of other substrates by human γ‐secretase.